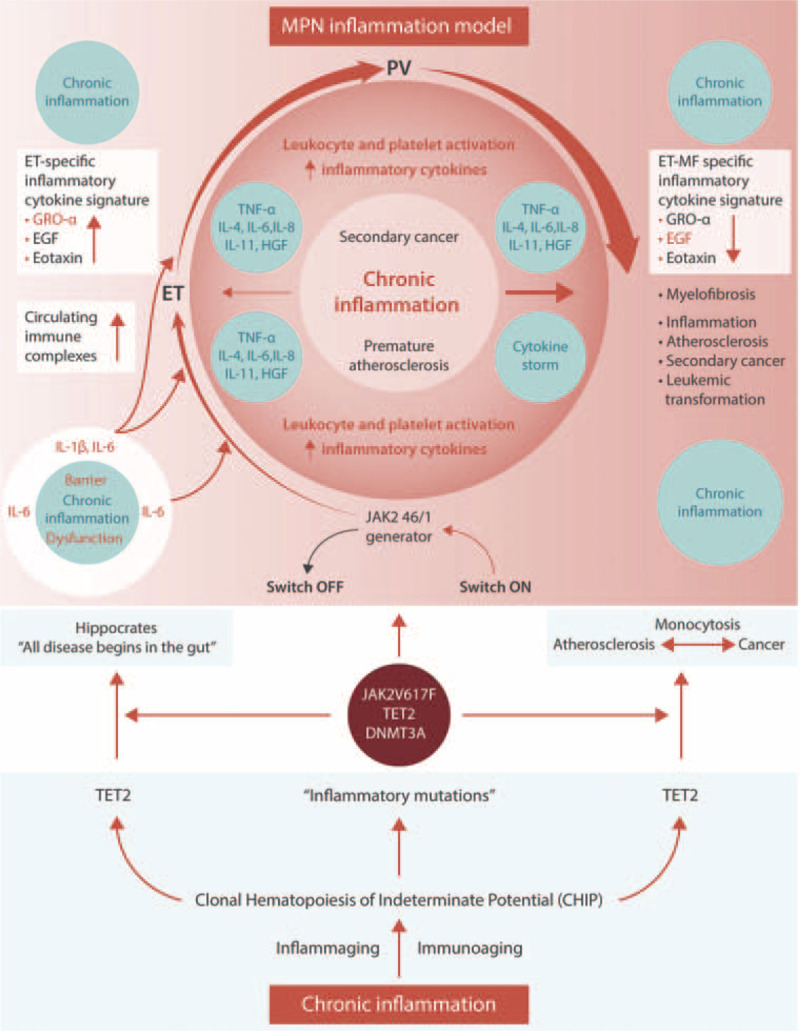# Correction Notice: Cytokine Profiling as a Novel Complementary Tool to Predict Prognosis in MPNs?

**DOI:** 10.1097/HS9.0000000000000482

**Published:** 2020-08-21

Since the publication of the article entitled “Cytokine Profiling as a Novel Complementary Tool to Predict Prognosis in MPNs?” (*HemaSphere.* 2020;4:e407), the author made grammar- and style-based corrections to the text as well as to Figure 1. These adjustments have been made and do not affect the outcome of this publication.

These additions have been made online: https://journals.lww.com/hemasphere/Fulltext/2020/06000/Cytokine_Profiling_as_a_Novel_Complementary_Tool.17.aspx

**Figure:**